# Effects of probiotics on child growth: a systematic review

**DOI:** 10.1186/s41043-015-0010-4

**Published:** 2015-05-02

**Authors:** Ojochenemi J Onubi, Amudha S Poobalan, Brendan Dineen, Debbi Marais, Geraldine McNeill

**Affiliations:** Public Health Nutrition Group, Institute of Applied Health Sciences, University of Aberdeen, Polwarth Building, Foresterhill, Aberdeen, AB25 2ZD UK

**Keywords:** Probiotics, Child growth, Systematic review

## Abstract

**Background:**

Child undernutrition has short and long term consequence for both individuals and society. Previous studies show probiotics may promote child growth and have an impact on under-nutrition.

**Methods:**

A systematic review of the literature was carried out on three electronic databases to assess evidence. The outcome measured was change in weight or height. A narrative analysis was conducted due to heterogeneity of included studies.

**Results:**

Twelve studies were included in the review of which ten were randomised controlled trials. A total of 2757 children were included, with 1598 from developing countries. The studies varied in type and quantity of probiotics given, duration of interventions, characteristics of participants, setting and units of outcome measures. Overall, five studies found a positive effect of probiotics on child growth. All five were conducted in developing countries with four studies conducted in mostly under-nourished children and one in well-nourished children. No significant effect on growth was found in the seven studies that were conducted in developed countries.

**Conclusion:**

The limited evidence suggests that probiotics have the potential to improve child growth in developing countries and in under-nourished children. More research is needed to explore this further.

**Electronic supplementary material:**

The online version of this article (doi:10.1186/s41043-015-0010-4) contains supplementary material, which is available to authorized users.

## Background

In 2011, the World Health Organisation (WHO) estimated that globally, 115 million (18%) children under-five years of age were underweight and 178 million (28%) were stunted [1]. A quarter of all children in developing countries suffer from malnutrition [2] with the majority of them residing in Africa and Asia [1]. Under-nutrition in children under five years of age increases the risk of mortality and morbidity due to diarrhoea and increased risk of infections by an estimated 35% and 11% respectively [3]. It also leads to long-term consequences such as delay of educational, social and economic development [4]. There has been some progress in the reduction in the proportion of underweight children under five years of age in developing countries from 30% to 23% between 1990 and 2009 [5], however, not sufficient to meet the Millennium Development Goal to reduce the under-five mortality rate by two thirds by the year 2015 [5].

Infectious disease (particularly diarrhoeal disease) is one of the underlying causes of under-nutrition (both macro and micronutrient deficiencies) through different mechanisms [3]. These nutrients are essential for adequate child growth and development and continuous poor nutrition results in poor growth [3,4]. Child growth has been identified as an important indicator for measuring the nutritional status and health of populations [6].

The past decade has seen a new era in medical science with increased use of ‘probiotics’ for health benefits, especially in diarrhoeal diseases. Probiotics are defined as live organisms which have health benefits for the host if taken in adequate amounts [7]. There have been recent reviews published on the effects of probiotics in children with specific disease conditions such as acute infectious diarrhoea [8], antibiotic-associated diarrhoea [9], necrotizing enterocolitis in very low birth weight infants [10], childhood atopy, *Helictobacter pylori* infection and infantile colic [11].

Probiotics have been shown to reduce the risk of infections such as infectious diarrhoea [8,12,13] as well as the incidence and duration of upper respiratory tract infections [12,14]. Probiotics may improve child growth through the prevention of infections and micronutrient deficiencies as they have been shown to improve the absorption of certain nutrients (calcium, zinc and vitamin B12) [12,15] and reduce the risk of anaemia [16].

Probiotics have been ingested for centuries, as part of fermented food products [7] and they have been isolated from traditional fermented products such as fermented milk ‘wara’ in Nigeria [17] and ‘Kule naoto’ among the Maasai in Kenya [18]. Fermentation is widely practiced and accepted in many regions of the world, particularly in Africa and Asia where fermented foods form a significant portion of the diets of rural communities [19]. In many African countries, the fermentation process is used to prepare complementary foods and therefore fermented foods are important in infant and child nutrition [20]. The process of fermentation is economical [19] and the potential use of fermented food to improve infant and young child feeding was explored during a joint Food and Agriculture Organization (FAO) and WHO workshop held in 1995 [21].

Consequently, the use of locally grown/culturally acceptable probiotic products could be used to improve the growth of the children at a low cost and could be implemented at large scale to reach the target community [22]. In spite of this recognition, only two systematic reviews to date have investigated the effects of probiotics on weight gain [23,24]. Both reviews however, focused on specific probiotic strains in target populations i.e. the review by Steenhout *et al.* in 2009 assessed the effects of bifidobacterium lactis in children younger than six months [24] and Million *et al.* assessed effects of lactobacillus species on weight gain in animals and healthy humans [23]. The aim of this review is to add to the evidence of the effects of probiotics on child growth irrespective of age, type of probiotic bacteria or nutritional status of the children.

## Methods

Three electronic bibliographic databases *(Medline, Embase and Cochrane Library)* were systematically searched using a robust search strategy. Literature published between 1947 and July 2011 was searched with no language restrictions. The Medline strategy (Additional file 1) was modified for the other databases. The Medline and Embase searches were updated using the same search strategy on the 26th October 2012 to identify any recent studies. All study designs that looked at use of any probiotic product in well-nourished and under-nourished children were included in the review. MeSH (Medical Subject Headings) terms and text words for ‘probiotics’, and ‘fermented milk product’ were combined appropriately with terms for ‘growth’, ‘anthropometry’ and ‘children’ to identify relevant studies. Studies that looked at probiotic use for the management of a disease condition; in children who had a specific disease condition rather than the management of under-nutrition and those that targeted other population groups such as pregnant women and children with impaired growth at birth were excluded.

Abstracts were read by two independent reviewers (OO and AP) to identify relevant studies. Full text articles of potentially eligible studies that met the selection criteria were obtained. Initially, the papers were critically appraised by two independent reviewers (OO and AP) until high consistency between the reviewers was achieved, and thereafter by one reviewer (OO). Reference lists of all included studies and review articles identified by the search were also checked to identify other relevant studies. One French language paper was professionally translated to English. All studies were assessed for methodological quality using a modified Cochrane review quality assessment form [25]. The reviewers were not blinded to the authors, journals, country of publications, results and conclusions of the papers.

A data extraction form was designed using guidelines from the University of York Centre for Reviews and Dissemination (CRD) checklist, piloted and amended before being used by two independent reviewers (OO and AP) to extract the data from the papers [26]. As available data in the published papers was sufficient for the narrative analysis that was conducted in this review, authors of primary studies were not contacted for any further information. Reviewers consulted regularly with each other to discuss any inclusion queries as they arose. Outcome measures assessed were change in weight, length/height, head circumference, Body Mass Index (BMI) and mortality rate. A narrative synthesis was conducted as meta-analysis of the data could not be undertaken due to heterogeneity of the studies in terms of different probiotic preperations used, age range of the participants, the timing of measurement of outcome variables and the growth measurement units (g/day, z-scores) between studies.

### Ethical clearance

Ethical clearance was not required as this is a systematic review of literature, and anonymized data was used.

### Results of the literature search

**Figure 1:**
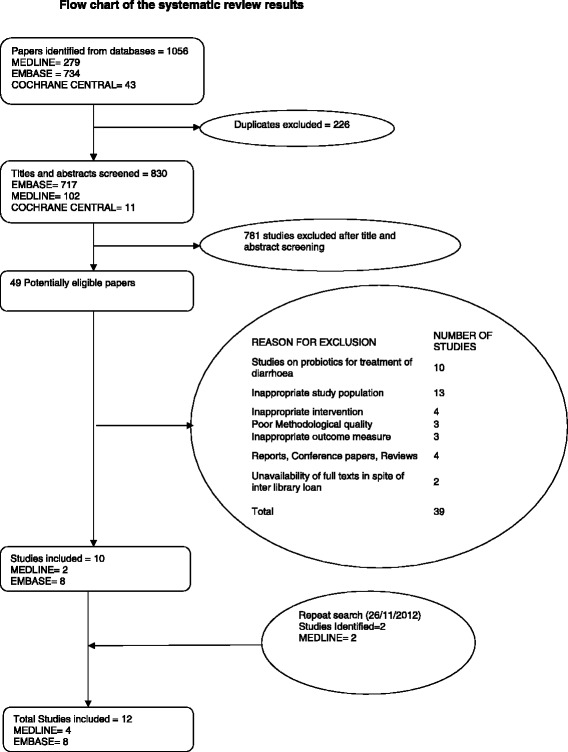
Flow chart of the systematic review results.

The initial systematic search identified 1056 citations, of which 49 potentially eligible articles were critically appraised. Ten studies met the inclusion criteria (Figure 1 – Prisma statement). The update search identified two recent relevant articles [27,28] giving a total of twelve studies to be included in this review.

Ten of the studies were randomised controlled trials (RCTs) [12,16,27-34] and two were non-randomised clinical controlled trials [13,35]. Five of the studies were conducted in developing countries in Asia [12,13,16,28,29]. The basic characteristics of the included studies are presented in Table 1. For this review we defined the study populations as ‘well-nourished’ if the anthropometric measurements showed that the majority of children were not stunted or wasted, and/or if the authors presented them as ‘healthy’; and as ‘under-nourished’ if the majority of children were underweight, stunted or wasted or if the authors presented them as ‘unhealthy’. Eight of the studies were conducted in well-nourished children [27,29-35] while four were conducted in under-nourished children [12,13,16,28]. All the studies in under-nourished children were conducted in developing countries while those on well-nourished children were conducted in developed countries except one study from Indonesia [29]. The age of the participants ranged from less than 28 days [30,31,33-35], to between one month and five years [12,13,16,27-29,32].

**Table 1:** **Basic characteristics of studies**

**Citation Country**	**Sample size(n)**	**Age/Gender: M/F**	**Description of intervention (I) and control (C) groups**	**Duration of intervention and Follow up**	**Outcome measures**
**Healthy children**					
Firmansyah *et al.* 2009 [29] Indonesia	n = 393	12 months	I: *Bifdobacterium longum* and *Lactobacillus rhamnosusin*	Duration:12 months	Weight gain per day and change in length measured between 12 months and 16 months
	I: 199	Gender: both (M/F):	200 ml Milk twice daily + prebiotics and LC-PUFA + Normal Diet	No Follow up	Other unrelated outcomes (motor and behavioural functions were measured at the end of the intervention)
	C: 194	I = 101/98	C: 200 ml Milk twice daily + Normal Diet with no probiotics	Measurements for weight gain taken after 4 months (16 months of age)	
		C = 102/92			
Scalabrin *et al.* 2009 [33] USA	n = 286	14 days	I: *Lactobacillus rhamnosus* in	Duration: from 14–120 days of age	Weight growth rate between 14 and 120 days of age
	I(a): 94	Gender: both	(a): Extensively hydrolyzed formula (EHF)	No follow-up	Length
	I(b): 98	(M/F):	(b): Partially hydrolyzed formula (PHF)		Head circumference
	C: 94	I(a): 50/44	C: EHF without probiotic		(*Length and head circumference measures were obtained at 30, 60, 90, 120, and 150 days of age)*
		I(b): 49/49	All children were exclusively formula fed and on demand		
		C: 44/50			
Saavedra *et al.* 2004 [32] USA	n = 131	3–24 months	I (High supplement (HS)): 1 x 10^7^ *Bifdobacterium lactis* Bb12 and *streptococcus thermophilus* CFU/g of standard milk based formula	Mean duration: 210 ± 127 days	Monthly weight and length
	I(HS): 44	Gender: both (M/F):	I (Low supplement(LS)): 1 x 10^6^ *Bifdobacterium lactis* Bb12 and *streptococcus thermophilus* CFU/g of standard milk based formula	No follow-up	
	I(LS): 43	I(HS): 22/17	C: Standard milk based formula with no probiotics		
	C: 44	I(LS): 21/19	Intake in each group had to be ≥ 240 ml/day for more than 14 days		
		C: 16/24			
Gibson *et al.* 2009 [30] Australia	n = 142	0–10 days	I: Bifdobacterium lactis 3 · 85 x 108 CFU+	Duration: 7 months	Weight gain per day, recumbent length, head circumference for 7 months, weight gain (g/d) from day 14 to day 119 (period of exclusively feeding the test formulas)
	I: 72	Gender: both	LC-PUFA(DHA) and AA in infant formula	No Follow up	
	C: 70	Intervention Female: 56%	C: infant formula		Others were BMI, and occurrence of adverse events
		Control			(Measurements conducted at approx. 2, 4, 6, 13, 17, 26, 30 weeks of age)
		Female: 53%	All children were exclusively formula fed but were allowed weaning from 4 months during which at least 500 ml/day of formula to be consumed		
Ziegler *et al*. 2003 [34] USA	n = 122	6–10 days	I(RP + P): Bifdobacterium lactis in reduced protein formula (RP)	Duration: Fed till 112 days of age (approximately 4 months of age)	Weight and length gain per day between 8-56 days, 56–112 days and 8–112 days
	I(RP + P): 40	Gender: both M/F ratio	I(RP): Reduced protein formula		
	I(RP): 40	Not reported	C: Normal protein formula	No Follow up	
	C: 42				
Puccio *et al*. 2007 [31] Italy	n = 138	Full term infants (<14 days)	I: 2 x 107 Bifdobacterium longum BL999 + 4 g/litre prebiotic in Infant formula	Duration: 7 months	Mean weight gain, recumbent length, head circumference at 14, 28, 56, 84 and 112 days of age
	I: 42	Gender M/F:	C: Formula without probiotics	No Follow up	
	C: 55	I: 20/22			
		C: 25/30			
Huet et al. 2006 [35] France	n = 203	1 – 28 days	I: Bifdobacterium lactis infant formula	Duration: 90 days	Daily weight gain, daily increase in height from day 0 to day 90
	I: 117	Gender: both M/F ratio not reported	C: Infant formula	No follow up	
	C: 86				
Gil-Campos et al. 2011 [27] Spain	N = 137	One month old infants	I: 107 cfu/g Lactobacillus fermentum CECT5716 + galactooligosaccharides (0.3 g/100 ml) in infant formula	Duration: 5 months	Average daily weight gain between baseline (one month) and 4 months of age
	I: 66	Gender M/F:	C: galactooligosaccharides (0.3 g/100 ml) in infant formula	No follow up	
	C: 71	I: 34/27			
		C: 38/22			
**Undernourished children**					
Sazawal *et al.* 2010 [16] India	n = 624	1–3 years	I: 1.9 x 10^7^ CFU per day of *Bifdobacterium lactis* HN019 + 2.4 g/day prebiotic in milk powder	Duration: One year	Weight gain at 6 months and 1 year
	I: 312	Gender: both M/F ratio not reported	C: milk powder	No follow-up	
	C: 312				
Saran *et al.* 2002 [13] India	n = 100	2–5 years	I: *Lactobacillus acidophilus* in curd (beet juice added) 1 x 10^8^ organisms/gm	Duration: 6 months	Body weight, height for 6 months
	I: 50	Gender: both	C: Isocaloric supplement (biscuits)	No follow up	Incidence of morbidity with respect to diarrhoea- frequency, severity and duration
	C: 50	Equal numbers			
He *et al.* 2005 [12] China	n = 402	3–5 years	I: *Thermophilus streptococci*, *Bulgaria lactobacilli* and *bifidum bacteria* in yogurt	Duration: 9 months	Body weight, height at 3,6 and 9 months
	I: 201	Gender: both	+ normal diet	No follow up	
	C: 201	M/F:	C: Normal Diet		
		I: 106/95			
		C: 111/90			
Surono *et al. *2011 [28] Indonesia	n = 79	15-54 months	I: 1 mg lyophilized *Enterococcus faecium* IS-27526 (2.31 x 10^8^ cfu/day) in 125 ml commercial UHT low fat milk	Duration: 90 days	Body weight
	I: 39	Gender: both		No follow up	
	C: 40	M/F:	C: 1 mg maltodextrin in 125 ml commercial UHT low fat milk		
		I: 17/22			
		C: 17/23			

NCHS: National centre for health statistics.

Probiotics were used in different combinations i.e. as a single probiotic [13,16,27,28,30,31,33-35] or multiple probiotics [12,29,32]; alone [12,13,27,28,32-35] or in combination with other products such as prebiotics [16,31] and long chain polyunsaturated fatty acids (LC-PUFA) [30] or both prebiotics and LC-PUFA [29]. Ten of the studies [16,27-35] compared a probiotic enriched formula/food/milk in the intervention group with a control group who had the same products but no probiotic added to it. One study compared probiotic food with no intervention (i.e. just a normal diet) in the other group [12] and another study compared probiotic enriched yoghurt with biscuits of the same caloric value [13].

The duration of supplementation with probiotics and timing of anthropometric measurements also varied across studies, from three months to one year. All the 12 included studies in this review investigated the effects of probiotics on growth in children. However, five of them measured the ‘difference in growth’ as their primary outcome by comparing children who were fed with probiotics with those who were not [12,13,16,28,29]. The seven other studies [27,30-35] measured the ‘safety and tolerance’ of probiotics in infant formula as their primary outcomes while measuring ‘growth’ as a secondary outcome.

## Results of the review

### Well-nourished children

Out of eight studies that were conducted among well-nourished children, only one study conducted in Indonesia, showed a significant difference in weight gain (0.93 g/day; p = 0.025) and weight-for-age (p = 0.036) between the intervention and control groups [29]. This was significantly higher than the growth standards recommended by the WHO [36] for that age group. The intervention group were given probiotics in addition to prebiotics and LC-PUFA and compared with a control group following a normal diet for a four month study period (Table 2, Section 1). Two major differences between this Indonesian study which showed improved weight gain and the other studies, are the settings of the studies and the age range of the participants. The children in the Indonesian study were older (aged 12 months and older) than the children in the other seven studies who were either less than 28 days of age [30,31,33-35], one month [27], or seven months of age [32]. With regards to the difference in settings, the study was conducted in a developing country (Indonesia) while the other seven studies were conducted in developed countries [27,30-35]. No significant improvements were seen in any of the other growth outcomes measured by height, head circumference or BMI.

**Table 2:** **Effects of probiotics on child growth**

***Section 1: In healthy children***								
**Author, year**	**Sample details**	**Outcomes and units of measurement**	**Results**					
**Country**								
**Type of study**								
**Quality**								
Firmansyah *et al.* 2009 [29]	**Intervention**:	**Outcome:**	**Outcome**	**Intervention**		**Control**	**Mean difference (CI)**	**p-value**
Indonesia	Age: 12 months	Weight, Length, Head circumference, Body Mass Index (BMI)	***Sample size***	***161***		***153***		
RCT	Sample size: 199		Weight (g/day)	7.57 ± 4.13		6.64 ± 4.08	0.93 (0.12-1.95)	0.025
**Quality:**	**Control:**	**Units of measurement:**	Change in weight-for-age	0.11 ± 0.40		0.02 ± 0.40	0.09 (0.01-0.18)	0.036
Unclear risk of bias for allocation concealment	Age: 12 months	*Weight:*	Weight (g)	9711 ± 1142		9643 ± 1218	Not reported	Not reported
	Sample size: 194	Weight gain (g/day)	Length (cm)	77.8 ± 3.0		77.9 ± 3.4	Not reported	Not significant
		Change in weight-for-age after 4 months	Head circumference (cm)	46.3 ± 1.3		46.4 ± 1.4	Not reported	Not significant
		Weight (g)	BMI (kg/m^2^)	16.0		15.9	Not reported	Not reported
		*Length*: Length after 4 months (cm)						
		*Head circumference*: Head circumference after 4 months (cm)						
		*BMI*: kg/m^2^						
Scalabrin *et al.* 2009 [33]	**Intervention:**	**Outcome**:	**Outcome**	**Intervention 1- EHF + P**	**Intervention 2 - PHF + P**	**Control EHF**	**Mean difference**	**p-value**
USA	Age: 14 days	Weight, Length, Head	***Sample size***	***63***	***77***	***70***		
RCT	Sample size:	circumference	Weight gain (g/day)	28.4 ± 0.67	26.8 ± 0.76	27.6 ± 0.72	Not reported	Not Significant
**Quality**:	-Extensively hydrolysed formula with probiotic (EHF + P): 94	**Units of measurement:**	Length (cm/day)	0.11 ± 0.002	0.11 ± 0.002	0.11 ± 0.002	No difference	
Low risk of bias for all parameters	-Partially hydrolysed formula with probiotic (PHF + P): 98	*Weight*:	Head circumference (cm/day)	0.05 ± 0.001	0.05 ± 0.001	0.05 ± 0.001	No difference	
		Weight gain (g/day)	ANOVA, 1-tailed t-tests					
	**Control:**	*Length*: change in length (cm/day)						
	Age: 14 days							
	Sample size: Extensively hydrolysed formula without probiotic (EHF): 94	*Head circumference*:						
		Change in head circumference (cm/day)						
Saavedra *et al.* 2004 [32]	**Intervention:**	**Outcome**:	**Outcome**	**Intervention 1 (HS)**	**Intervention 2 (LS)**	**Control**	**Mean difference**	**p-value**
USA	Age: 3–24 months	Weight and Height	**Sample size**	39	39	40		
RCT	Sample size:	**Units of measurement:**	Change in weight-for-age	0.09 ± 0.64	0.06 ± 0.72	0.16 ± 0.69	Not reported	Not significant
**Quality**:	-High Supplement probiotic in formula (HS): 39	*Weight*:	Change in weigh-for-length	0.40 ± 0.85	0.53 ± 1.10	0.45 ± 0.75	Not reported	Not significant
Unclear risk of bias in allocation concealment	-Low Supplement probiotic in formula (LS): 39	change in weight-for-age z-score	Change in height-for-age	−0.06 ± 0.44	−0.09 ± 0.60	−0.04 ± 0.59	Not reported	Not significant
	**Control- formula**	change in weight-for-length score						
	Age: 3–24 months	*Height*:						
	Sample size: 40	change in height- for-age z-score						
Gibson *et al.* 2009 [30]	**Intervention:**	**Outcome**:	**Outcome**	**Intervention**		**Control**	**Mean difference**	**p-value**
Australia	Age: <10 days	Weight, Length, Head Circumference, BMI	**Sample size**:	62		62		
RCT	Sample size: 72	**Units of measurement**:	Weight gain (g/day)	M(24) 33 · 6 ± 7 · 5		M(19) 31 · 6 ± 7 · 7	1.5 (−0.08-3.1)	Not significant
**Quality**:	**Control:**	*Weight* : Weight gain (g/day)		F(31) 28 · 1 ± 5 · 8		F(24) 26 · 5 ± 4 · 9		
Low risk of bias in all parameters	Age: <10 days Sample size: 70	*Length:* Length gain (mm/month)	Length gain (mm/month)	M(24) 35 ± 3 · 7		M(19) 37 · 3 ± 4 · 9	Not reported	Not significant
		*Head circumference*: Change in head circumference (mm/month)		F(27) 32 · 8 ± 4		F(23) 32 ± 4 · 6		
		*BMI*: change in BMI per month (kg/cm^2^/month)	Head circumference (mm/month)	M(23) 18 ± 2 · 4		M(19) 17 · 5 ± 3 · 4	Not reported	Not significant
				F(29) 16 · 1 ± 2 · 7		F(24) 16 ± 3		
			BMI (kg/cm^2^/month)	M(24) 1 · 1 ± 0 · 6		M(19) 1 ± 0 · 5	Not reported	Not significant
				F(27) 0 · 9 ± 0 · 5		F(23) 0 · 8 ± 0 · 4		
			ANOVA correcting for sex					
Zeigler *et al.* 2003 [34]	**Intervention:**	**Outcome**:	**Outcome**	**Intervention (RP + P)**	**Intervention (RP)**	**Control**	**Mean difference**	**p-value**
USA	Age: 6–10 days	Weight and Height	**Sample size**	28	27	C:33		
RCT	Sample size:	**Units of measurement**:	Weight gain (g/day)	28.13 ± 4.63^§^	29.3 ± 5.41^§^	31.05 ± 5.88^§^	Not Reported	0.229
**Quality**:	RP + P	*Weight*: g/day						
The risk of bias in adequate sequence generation, allocation concealment and blinding was unclear while there was a high risk of bias in reporting of incomplete outcome data	(*Bifidobacterium lactis* in reduced protein formula): 40	*Length:* mm/day		M 13 32.1 ± 5.2	M 8 32.0 ± 4.7	M 19 32.2 ± 5.2		
				F 15 24.7 ± 4.9	F 19 28.2 ± 5.8	F 14 29.5 ± 6.9		
	RP (Reduced protein formula): 40		Length gain (mm/day)	M 13 1.14 ± 0.11	M 8 1.14 ± 0.09	M19 1.16 ± 0.09	Not reported	0.377
				F 15 1.02 ± 0.07	F 19 1.06 ± 0.10	F14 1.07 ± 0.14		
	**Control:**							
	Age: 6–10 days							
	Sample size							
	Normal protein formula: 42							
Puccio *et al.* 2007 [31]	**Intervention:**	**Outcomes**:	**Outcome**	**Intervention**		**Control**	**Mean difference (90% CI)**	**p-value**
Italy	Age: <14 days	Weight, height, head circumference	**Sample size**	42		55		
RCT	Sample size: 65	**Units of measurement:**	Weight (g/day)	Not reported		Not reported	0.50 (−1.48 ± 2.48)	Not reported
**Quality**: Risk of bias was unclear in both adequate sequence generation and allocation concealment	**Control:**	*Weight*: weight gain (g/day)	Height (mm/month)	M 35.1 ± 4.2		M: 35 ± 4.4	Not reported	0.1
	Age: <14 days	*Height*: change in height (mm/month)		F 32.2 ± 4.3		F : 32.2 ± 4.6		0.1
	Sample size: 69	*Head circumference*: Change in head circumference (mm/month)	Head circumference (mm/month)	M: 17.9 ± 2.7		M : 17.4 ± 2.9	Not reported	>0.1 for all
				F: 16.0 ± 2.8		F: 15.5 ± 3.0		
Huet *et al.*, 2006 [35]	**Intervention:**	**Outcomes**:	**Outcome**	**Intervention**		**Control**	**Mean difference**	**p-value**
France	Age: 1–28 days	Weight, Height, Head circumference	**Sample size**	117		86		
CCT	Sample size: 117	**Units of measurement**:	Weight gain (g/day)	29.6 ± 6.6		29.8 ± 6.3	Not reported	Not significant
**Quality**: The study had high risk of bias in adequate sequence generation, allocation concealment and blinding.	**Control:**	*Weight*: weight gain (g/day)	Height (cm/day)	0.110 ± 0.018		0.111 ± 0.018	Not reported	Not significant
	Age: 1-28 days	*Height:* height gain (cm/day)	Head circumference(mm/day)	0.56 ± 0.12		0.55 ± 0.12	Not reported	Not significant
	Sample size: 86	*Head circumference:* change in head circumference (mm/day)						
Gil-Campos *et al.* 2011 [27]	**Intervention:**	**Outcomes:**	**Outcome**	**Intervention**		**Control**	**Mean difference**	**p-value**
Spain	Age: 1 month	Weight, Height, Head Circumference	**Sample size**	61		60		
RCT	Sample size: 71	**Units of measurement:**	Weight gain (g/day)	24.8 ± 5.1		25.3 ± 6.0	Not reported	Not significant
Quality: There was low risk of bias in all parameters.	**Control:**	*Weight*: weight gain (g/day), weight at 6 months (kg), weight-for-age z-scores at 6 months	Length gain (mm/day)	0.96 ± 0.3		0.90 ± 0.2	Not reported	Not significant
	Age: 1 month	*Length*: Length gain (mm/day), Length at 6 months (cm), Length for age z-scores at 6 months	Head Circumference (mm/day)	0.43 ± 0.1		0.421 ± 0.1	Not reported	Not significant
			Weight at 6 months (kg)	8.0 ± 0.9		7.9 ± 1.0	Not reported	Not significant
	Sample size: 66	*Head Circumference*: Head Circumference at 6 months (cm), Head circumference z-scores at 6 months	Length at 6 months (cm)	68.1 ± 3.4		66.6 ± 2.5	Not reported	0.038
			Head Circumference at 6 months (cm)	43.7 ± 1.6		43.7 ± 1.3	Not reported	Not significant
			Weight for age z-scores at 6 months	Not reported		Not reported	Not reported	p = 0.061
			Length for age z-scores at 6 months	Not reported		Not reported	Not reported	p = 0.021
			Head circumference z-scores at 6 months	Not reported		Not reported	Not reported	p = 0.453
***Section 2: In under-nourished children***								
**Author, year**	**Sample details**	**Outcomes and units of measurement**	**Results**					
**Country**								
**Type of study**								
**Quality**								
**Nutritional status**								
Sazawal *et al.* 2010 [16] India	**Intervention:**	**Outcomes:**	**Outcome**	**Intervention**		**Control**	**Mean difference**	**p-value**
RCT	Age: 1–3 years	Weight, height	**Sample size**	**257**		**245**		
**Quality**: The risk of bias was low for all parameters	Sample size: 312	**Units of measurement:**	Weight gain (g/year)	2,130 ± 590		2,000 ± 590	130 (30–230)	0.02
None severely malnourished	**Control:**	*Weight*: weight gain (g/year), change in weight for age z-score	Change in weight-for-age z-score	0.34 ± 0.54		0.26 ± 0.54	0.08 (−0.02 to 0.17)	0.12
**Nutritional status**								
Normal	Age: 1–3 years		Height (cm/year)	8.49 ± 1.41		8.28 ± 1.35	0.20 (−0.04 to 0.45)	0.09
I: 107 (34.3%) C: 95 (30.4%)	Sample size: 312	*Height*: height gain (cm/year), change in height for age z-score after one year	change in height for age z-score after 1 year	0.21 ± 0.42		0.18 ± 0.49	0.03 (−0.06 to 0.10)	0.55
Wasted			Difference in weight/height	0.44 ± 0.65		0.34 ± 0.63	0.09 (−0.01 to 0.21)	0.09
I: 15 (4.8%) C: 14 (4.5%)								
Stunted								
I: 137 (43.9%) C: 157 (50.3%)								
Wasted and stunted								
I: 53 (17.0%) C: 46 (14.7%)								
Saran *et al.*, 2002 [13]	**Intervention:**	**Outcomes**:	**Outcome**	**Intervention**		**Control**	**Mean difference**	**p-value**
India	Age: 2–5 years	Weight, height	**Sample size**	**50**		**50**		
Non-randomised controlled trial	Sample size: 50	**Units of measurement:**	Weight (g/6 months)	1,290 ± 730		810 ± 840	0.002	Not reported
**Quality**: high risk of bias for adequate sequence generation, allocation concealment and blinding.	**Control:**	*Weight*: weight gain (g per 6 months)	Height: (cm/6months)	3.21 ± 1.48		1.74 ± 0.80	Not reported	0.0001
**Nutritional status**								
Stunted (height for age) and matched in both groups	Age: 2–5 years	*Height:* height gain (cm per 6 months)						
	Sample size: 50							
He *et al.*, 2005 [12]	**Intervention:**	**Outcomes**:	**Outcome**	**Intervention**		**Control**	**Mean difference**	**p-value**
China	Age: 3–5 years	Weight, height	**Sample size**	**201**		**201**		
RCT	Sample size: 201		Gram per 3, 6 and 9 months	700 ± 430		490 ± 350	Not reported	0.01
**Quality**:	**Control:**	**Units of measurement:** *Weight*: Weight gain (g per 3, 6 and 9 months), Change in weight-for-age at 3, 6 and 9 months		980 ± 620		800 ± 600		0.01
There was an unclear risk of bias in adequate sequence generation and high risk of bias in both allocation concealment and blinding	Age: 3–5 years			1,420 ± 760		1,200 ± 670		0.01
	Sample size: 201		Change in weight-for-age at 3, 6 and 9 months	0.139 ± 0.228		0.031 ± 0.184		0.01
**Nutritional status**								
Undernourished - weight for age and/or height for age were below reference values		*Height*: change in height for age z-scores at 9 months		0.058 ± 0.306		−0.047 ± 0.28		0.01
				0.078 ± 0.365		−0.043 ± 0.28		0.01
			Change in height for age z-scores at 9 months	0.123 ± 0.168		0.077 ± 0.175	Not reported	<0.01
Surono et al. 2011 [28] Indonesia	**Intervention:**	**Outcomes:** Weight	**Outcome**	**Intervention**		**Control**	**Mean difference**	**p-value**
RCT	Age: 15–54 months	**Units of measurement:**	**Sample size**	37		39		
	Sample size: 39	*Weight:* Mean gain in bodyweight after 90 days	Mean bodyweight gain (g)	1280 ± 940		990 ± 990	Not reported	Not reported
**Quality:**	**Control:**							
There was an unclear risk of bias in adequate sequence generation, allocation concealment and blinding.	Age: 15–54 monthss							
**Nutritional status**								
Underweight	Sample size: 40							
I: 20 C: 20								
Severe Underweight								
I: 7 C:10								
Normal Bodyweight								
I:10								
C:9								

No baseline differences between groups; Values presented in mean ± SD unless specified; NHCS: National Health Centre Statistics; MUAC: Mid Upper Arm Circumference.

^§^The results of weight gain per day for both sexes were combined and presented by the authors.

### Under-nourished children

Four studies were conducted among under-nourished children between the ages of one and five years [12,13,16,28]. All four studies were conducted in developing countries. In two of these studies, all the children were under-nourished [12,13], while in the remaining two studies there was a mixture of children who were normal weight, underweight, stunted and/or wasted (Table 2, Section 2) [16,28]. All four studies found improved weight in the probiotic group compared with the control group. Three studies showed increased weight gain in grams after six (1290 ± 730 vs 810 ± 840) [13], nine (1420 ± 760 vs 1200 ± 670) [12], and 12 (2130 ± 590 vs 2000 ± 590) [16] months of supplementation in the probiotic groups compared with the control groups respectively. However, the mean differences were not reported in any of the studies. He *et al.* [12] also noted significant increases in change in weight-for-age z-scores. In the fourth study by Surono *et al.* [28], the mean weight gain of mostly under-nourished children in the probiotic group was 1280 ± 940g compared with the children in the control group with mean weight gain of 990 ± 990 g. This difference became significant when the results were stratified by nutritional status (normal weight, underweight and severly underweight) as children with normal body weight in the probiotic group weighed significantly more than those in the control group. Regarding the other growth outcomes, two studies found a significant difference in height of the children [12,13]. In He *et al.* [12], the children in the probiotic group had a change in height-for-age z-score of 0.123 ± 0.168 while those in the control group had a change in height-for-age z-score of 0.077 ± 0.175 at nine months of supplementation (p < 0.01). Again, this increase in height-for-age z-scores in the probiotic group was significantly higher than the reference value recommended by the WHO for children of that age group while in the control group, the change was less than the WHO reference value [36]. The other study by Saran *et al.* [13] showed that, after 6 months, the children in the probiotic group grew an average of 3.21 ± 1.48 centimetres in length compared to the control group 1.74 ± 0.80cm (p = 0.0001).

## Discussion

This review found a benefit of dietary intake of probiotics in weight and length/height gain, potentially in children who are under-nourished and also healthy children living in developing countries. In clinics worldwide, the WHO growth charts are used for monitoring the growth of children in relation to that of the expected value for age [36]. Two out of the five studies [12,29] that showed significant improvement in growth, noted that the children in the probiotic groups had growth curves that were significantly higher than [12] or closer to [29] the WHO reference value than the children in the control groups. One other notable finding in one study [12] is the improvement in height-for-age z-scores in children who took probiotics compared to those in the control group. Change in height-for-age z-scores indicates catch-up growth in children [37], therefore, probiotics may help in promoting compensatory growth of children with stunted growth [3]. The effect of probiotics on the growth of under-nourished children was also investigated in a large RCT (PRONUT study) by Kerac *et al.* [38]. In this study, probiotics did not seem to confer any benefits on the health or the nutritional status of these children. However, compared to the other studies conducted in undernourished children in this review who were community living and suffered from chronic undernutrition, the children in the PRONUT study were acutely malnourished and needed hospital admission, almost half were HIV positive and all the children were on antibiotics. The lack of effects in the PRONUT study could have been confounded by the fact that the children were HIV positive, on antibiotics and acutely malnourished.

No evidence was found for a benefit of dietary intake of probiotics on growth in well-nourished children in developed countries. Some benefit was shown in terms of weight gain in the one study in well-nourished children in a developing country [29]. The benefit shown in this study as compared to the others in well-nourished children may be due to various factors including the addition of prebiotics and LC-PUFA with the probiotics, the age of the children and/or the developing country setting. While some studies have shown there could be a synergistic effect when combining pre- and probiotics and a modulation of the immune system by combining probiotics with LC-PUFA [7], other studies in this review that also added either prebiotics or LC-PUFA did not show any significant benefits in developed country settings [30,31]. This indicates that the differences in regimens are probably not responsible for the difference in findings. The fact that this study was conducted on an older group of children (12 months of age compared to the other children who were less than 28 days at start of study) might be another likely reason for the differences found. Findings in the review by the European Society for Paediatric Gastroenterology, Hepatology and Nutrition (ESPGHAN) indicate that probiotics administered to children younger than four months of age do not lead to any consistent clinical effects such as reduction of gastro-intestinal infections unlike when given beyond early infancy [39]. Most children in the other studies of well-nourished children were younger than four months old, which highlights the need for further research on older children. Furthermore, the difference in benefits to growth may be related to a number of factors peculiar to developing country settings. It is worth noting that environmental factors such as the diet, eating practices and sanitation may affect the efficacy of probiotics by modifying the commensal gut flora [14], hence need to be taken into consideration while advocating the use of probiotics in different settings. Only one study was conducted among well-nourished children in developing country making it difficult to generalise the results for healthy children in both developed and developing country settings. Therefore, more research is needed particularly among healthy children in developing countries for effective comparison. In addition, only published studies were included in this review which introduces bias and limiting the evidence.

Although this review did not aim to assess the benefits of specific probiotic strains on diferent populations, it is worth noting that B*ifidobacterium lactis* HN019; *Bifdobacterium longum and Lactobacillus rhamnosus; lactobacillus acidophilus; thermophilus streptococci*, *bulgaria lactobacilli* and *bifidum bacteria; and enterococcus faecium* IS-27526 were all highlighted as beneficial strains in general by the included studies. Different types of probiotics have distinct effects and even those strains that are closely related may have different clinical effects [7,8]. There is an emphasis by the Food and Agricultural Organization (FAO) of the United Nations that the effects of a specific strain should not be assumed to occur in other strains [7]. More research is needed on the specific strains that improve growth in children in developing countries.

Probiotic containing food used in the studies in developing countries were from the local markets [12] or locally prepared probiotics [13,28]. Given the benefits of probiotics on child growth as hightlighted in this review, use of readily available and less expensive fermented food products as a vehicle of probiotics might play an important role in improving nutrition, treating enteric infections [40] and promoting compensatory growth in children in developing countries through these different mechanisms. However, more research is needed into the consumer confidence, acceptability of fermented products as a source of probiotics and also the safety aspects before promoting fermented foods in complementary feeding in developing countries. Although the administration of probiotics was not associated with serious adverse effects from any of the studies included in this review, it is recommended that probiotics be given to critically ill or immuno-suppressed children with caution as there have been rare cases of probiotic infections in immuno-suppressed individuals and people with indwelling catheters [8]. In spite of some probiotics studies [38] showing no difference in probiotic related sepsis among acutely malnourished and immunocompromised children, the dearth of information on the safety issue of probiotics in malnourished children should be considered before promoting probiotics in this specific population.

### What is already known and what this review adds

Previous reviews have shown the effectiveness of probiotics on growth in children with specific disease conditions, whereas this is the first to report on the effects of probiotics on measures of child growth in non clinical settings. It is important to note that due to the paucity of the number of studies that assessed the effects of probiotics on child growth, all studies regardless of the vehicle used in administering the probiotic were included. Usually probiotics are added to infant formulas in order to modify the micro-biota of babies who are not breastfed to make it on par with breast-fed infants [24,39], who benefit from certain lactic acid bacteria and indigestible oligosaccharides which enhance the proliferation of probiotics [7,11]. Although a number of studies using probiotic-enriched formula were included in this review, the results by no means promote infant formula fortified with probiotic as a substitute for breast milk, as exclusive breastfeeding in the first six months is a key child survival strategy [41,42]. This review showed that probiotics improves growth in children and highlighted that these benefits were more significiant in under-nourished children and in a developing country setting while highlighting no adverse effects on children [27-29,32,33]. Given that under-nutrition is more prevalent in developing country settings [3], this review suggests that probiotics may play an important role in improving nutrition, promoting compensatory growth in low resource countries. In addition, it also argues for the idea of exploring the use of locally available and culturally acceptable fermented products as a vehicle of probiotics, by investigating the safety and acceptability of the products [40].

## Conclusion and recommendations

This review found a benefit of dietary intake of probiotics in terms of weight and height gain in under-nourished children and possible benefit in terms of weight gain in well-nourished children in developing countries. It is suggested that the supplementation promotion of locally available foods with probiotics could be an effective intervention to improve growth in children, especially in developing countries. Further research is needed to investigate this benefit among well-nourished children in a developing country context especially in Africa where limited evidence is available; under-nourished children in a developed country context, as well as in older children. Future studies on probiotics should measure growth as a primary outcome to strengthen the evidence and explore the acceptabilty of the use of fermented milk products as a vehicle for probiotics.

## Additional file

Additional file 1:
**Medline Search Strategy.**
