# Supplementary material for: Effects of probiotics on child growth: a systematic review
Source: J Health Popul Nutr. 2015 May 2;34:8. doi: 10.1186/s41043-015-0010-4 (PMC5025996; doi:10.1186/s41043-015-0010-4)
Supplement: Additional file 1: — Medline Search Strategy. [file 41043_2015_10_MOESM1_ESM.doc]

Additional file 1: MEDLINE SEARCH STRATEGY

|  | **Searches** |
| --- | --- |
| 1 | exp Probiotics/ |
| 2 | exp Cultured Milk Products/ |
| 3 | probiotic$.mp. |
| 4 | ((ferment$ or cultured) adj3 milk adj1 product$).mp. |
| 5 | or/1-4 |
| 6 | exp Child/ |
| 7 | child$.mp. |
| 8 | exp Child, Preschool/ |
| 9 | exp Infant/ |
| 10 | infant$.mp. |
| 11 | (paediatric$ or pediatric$).mp. |
| 12 | or/6-11 |
| 13 | exp Growth/ |
| 14 | exp Growth Charts/ |
| 15 | exp Anthropometry/ |
| 16 | exp Body Weight/ |
| 17 | growth.mp. |
| 18 | anthropometr$.mp. |
| 19 | exp Body Height/ |
| 20 | (stunt$ adj5 child$).mp. |
| 21 | (body adj1 (weight or height)).mp. |
| 22 | or/13-21 |
| 23 | **5 and 12 and 22** |
